# A bioinformatics pipeline for *Mycobacterium tuberculosis* sequencing that cleans contaminant reads from sputum samples

**DOI:** 10.1371/journal.pone.0258774

**Published:** 2021-10-26

**Authors:** Betzaida Cuevas-Córdoba, Cristóbal Fresno, Joshua I. Haase-Hernández, Martín Barbosa-Amezcua, Minerva Mata-Rocha, Marcela Muñoz-Torrico, Miguel A. Salazar-Lezama, José A. Martínez-Orozco, Luis A. Narváez-Díaz, Jorge Salas-Hernández, Vanessa González-Covarrubias, Xavier Soberón

**Affiliations:** 1 Laboratorio de Farmacogenómica, Instituto Nacional de Medicina Genómica (INMEGEN), Ciudad de México, México; 2 Instituto de Investigaciones Biológicas, Universidad Veracruzana, Xalapa, Veracruz, México; 3 Departamento de Desarrollo Tecnológico, Instituto Nacional de Medicina Genómica (INMEGEN), Ciudad de México, México; 4 Clínica de Tuberculosis y Enfermedades Pleurales, Instituto Nacional de Enfermedades Respiratorias (INER), Ciudad de México, México; CNR, ITALY

## Abstract

Next-Generation Sequencing (NGS) is widely used to investigate genomic variation. In several studies, the genetic variation of *Mycobacterium tuberculosis* has been analyzed in sputum samples without previous culture, using target enrichment methodologies for NGS. Alignments obtained by different programs generally map the sequences under default parameters, and from these results, it is assumed that only *Mycobacterium* reads will be obtained. However, variants of interest microorganism in clinical samples can be confused with a vast collection of reads from other bacteria, viruses, and human DNA. Currently, there are no standardized pipelines, and the cleaning success is never verified since there is a lack of rigorous controls to identify and remove reads from other sputum-microorganisms genetically similar to *M*. *tuberculosis*. Therefore, we designed a bioinformatic pipeline to process NGS data from sputum samples, including several filters and quality control points to identify and eliminate non-*M*. *tuberculosis* reads to obtain a reliable genetic variant report. Our proposal uses the SURPI software as a taxonomic classifier to filter input sequences and perform a mapping that provides the highest percentage of *Mycobacterium* reads, minimizing the reads from other microorganisms. We then use the filtered sequences to perform variant calling with the GATK software, ensuring the mapping quality, realignment, recalibration, hard-filtering, and post-filter to increase the reliability of the reported variants. Using default mapping parameters, we identified reads of contaminant bacteria, such as *Streptococcus*, *Rhotia*, *Actinomyces*, and *Veillonella*. Our final mapping strategy allowed a sequence identity of 97.8% between the input reads and the whole *M*. *tuberculosis* reference genome *H37Rv* using a genomic edit distance of three, thus removing 98.8% of the off-target sequences with a *Mycobacterium* reads loss of 1.7%. Finally, more than 200 unreliable genetic variants were removed during the variant calling, increasing the report’s reliability.

## Introduction

Tuberculosis (TB) is a communicable disease caused by the bacillus *Mycobacterium tuberculosis*. This disease has gained global importance for several decades due to its re-emergence, treatment, and control complexity. Since 2012, TB has been the leading cause of death by a single infectious agent and the 9th cause of general death worldwide [1]. Next-Generation Sequencing (NGS) has increased the knowledge of TB in aspects such as genetic diversity, transmission chains, discrimination between relapse or re-infection, surveillance, and drug resistance [2, 3]. Despite NGS costs decrease [4, 5], it has not been clinically implemented, in part due to two key bottlenecks; one is the direct use of clinical samples without previous culture, and the other is the availability of fast and reliable bioinformatics tools [6, 7].

Clinical samples comprise thousands of genetically diverse microorganisms, some of which share remarkable similarities in their sequences [8]. Analysis of these samples by NGS can be performed by whole-genome or by panels examining selected genes. The approach based on custom panels and the subsequent enrichment of the desired target regions has the advantages of broader coverage of specific regions of interest, easier multiplexing of samples, lower cost, and less complex analysis. Capture by hybridization is one of the major target enrichment technologies to achieve uniform coverages of sequences and good reproducibility [9].

Despite these advantages, most NGS studies on TB utilize DNA extracted from mycobacterial cultures [10–12], delaying the result by several weeks, while the minority do so directly from sputum [13–15]. However, the strategy to filter reads from microorganisms other than *M*. *tuberculosis* from clinical samples is not fully standardized [16]. General mapping practices currently align NGS reads to a reference genome, assuming that only reads of the microorganism of interest will remain. However, the presence or absence of other microorganism reads is not evaluated.

An optimized bioinformatics pipeline that guarantees the report of *M*. *tuberculosis* sequences exclusively from a complex biological matrix is required; lack of stringency in this analysis could generate erroneous variant reports. Thus, a specific software such as SURPI (sequence-based ultrarapid pathogen identification) [17] is required for taxonomic identification of microorganisms by NGS, which should be a critical first step before variant calling. In addition, generating a reliable list of genetic variants requires the inclusion of quality controls to eliminate sequencing errors such as amplification bias, software errors, mapping artifacts, and variant recalibration. The Genome Analysis Toolkit (GATK) pipeline includes parameters to control these errors, and the documentation regarding best practice recommendations is frequently updated [18–21].

Several quality filtering steps could be implemented when high depth sequencing (>1000X) is used, which eliminates off-target sequences, keeping enough *M*. *tuberculosis* reads, and reporting highly reliable variants. Therefore, this study aimed to design a bioinformatic pipeline based on SURPI and GATK to identify *M*. *tuberculosis* reads and remove those from untargeted microbial contamination from sputum samples. This bioinformatic proposal improves the quality of *M*. *tuberculosis* variant calling of NGS reads obtained directly from sputum samples, facilitating its implementation as a diagnostic tool and drug selection.

## Materials and methods

### Sputum samples and DNA extraction

We recruited 36 patients with TB and two negative controls (without TB) from the National Institute of Respiratory Diseases (INER) in Mexico City, Mexico. The project was approved by”Comité de Ética en Investigación del Instituto Nacional de Medicina Genómica”, approval number CEI 2017/21. All patients signed the informed written consent and donated a sputum sample in compliance with the Helsinki Declaration.

Microbiology sputum smears and cultures were performed on the samples. We obtained 26 subjects with strongly positive smears (3+), two with moderately positive smears (2+), five with weakly positive smears (1+), and three with negative smears and subsequent positive culture (0+). Microbiological cultures confirmed 28 *Mycobacterium tuberculosis* complex, four *Mycobacterium tuberculosis*, and four *Mycobacterium bovis* samples. Sputum samples were heat-inactivated at 80°C for 20 min and pretreated with 500 mg of N-acetyl-L-cysteine for 15 min, followed by two volumes of NaOH 2.0% for 10 min with two intermediate agitations. PBS was added to bring the mixture to a final volume of 50 ml, then centrifuged. The pellet was resuspended with 10 ml of deionized sterile water, incubated for 30 min, centrifuged, and resuspended in 1.5 ml of water. DNA extraction was performed with some modifications to the method of Warren and col [22] to favor DNA isolation from *Mycobacterium*.

### Targeted enrichment of genomic DNA regions and NGS

A custom gene capture design was performed by Agilent design services (Agilent Technologies, USA), selecting 174 gene regions of the *M*. *tuberculosis* genome related to virulence and drug resistance (panel size 244 kbp).

According to Agilent’s Sure Select XT kit protocol, sequencing libraries were prepared using 200 ng of total DNA for each sample. Libraries quality was assessed with a Tape Station (Agilent), verifying fragment sizes of 225 to 275 base pairs (bp). Hybridization and capture were conducted with RNA probes, and targeted regions were purified with magnetic beads, followed by post-hybridization amplification for indexing. The NGS was performed using an Illumina NextSeq platform (2 × 150 bp) in pair-end reads.

### Design of the bioinformatic strategy

**Fig 1:**
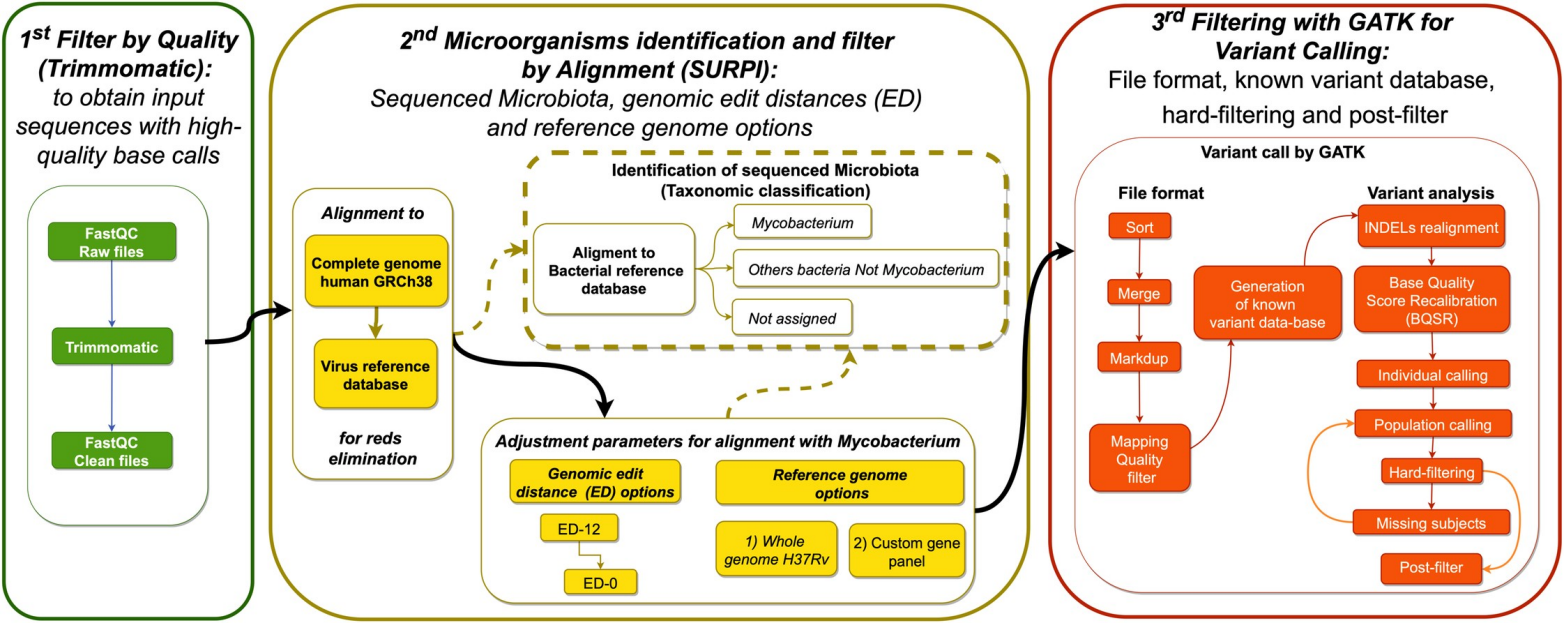
Bioinformatic pipeline. The workflow comprises three steps: 1) pre-processing, 2) microorganisms’ identification and filtering by alignment, and 3) high-quality variant calling. The dotted line represents the steps for taxonomic classification before and after mapping with the reference genomes.

We designed a specific pipeline to analyze the sequencing data from sputum samples, based mainly on two open-source genomic tools SURPI and GATK. The pipeline aimed to generate a report of *M*. *tuberculosis* genetic variants, minimizing the false-positive results (Fig 1). This pipeline consists of three steps: 1) Ensuring input files with highly accurate base calling. 2) Alignment and taxonomic classification of all reads to filter contaminant sequences, following by mapping with different reference genomes and genomic edit distance values to identify *M*. *tuberculosis* sequences. 3) Reliable variant calling by conducting INDELs realignment, base quality score recalibration (BQSR), a hard-filtering for SNPs and INDELs, and a final post-filter.

#### 1st step: Filtering input sequences with high-quality base calls

The FASTQ files were analyzed with FastQC during the pre-processes (http://www.bioinformatics.babraham.ac.uk/projects/fastqc/). Adapters and overrepresented sequences were eliminated with the Trimmomatic software [23]. Then, a sliding window was used to obtain high-quality sequences, considering five bases with an average quality value ≥ 28 and a minimum length of 70 bp per read. Furthermore, K-mers were eliminated considering a fixed distance according to the FastQC results (Fig 1).

#### 2nd step: Microorganism identification and filtering by alignment with SURPI

This step used SURPI (fast mode) as a taxonomic classifier to identify and filter unwanted reads by serial alignments. SURPI is a free bioinformatics tool for pathogen identification that can process complex metagenomic data and broadly classify reads for viruses and bacterial genomes. This software executes an accelerated analysis, scanning data of over 500 million reads in a maximum of 5 h due to two state-of-the-art aligners: SNAP and RAPSearch. The SNAP alignment tool also offers more accurate bacterial detection than BWA and Bowtie 2. Relative to BLASTn, it has a slight reduction in sensitivity (99.5% vs. 100%) but higher specificity (98.5% vs. 97.7%) [17].

Initially, we aligned all high-quality reads of FASTA files with the genome GRCh38 to eliminate human reads and then with the SURPI virus database to discard virus reads, obtaining BAM filtered files. The BAM files were aligned with the SURPI bacterial reference database to obtain the taxonomic classification of the sequenced microbiota (Fig 1). The taxonomic classification was used to quantify the number of reads identified as *Mycobacterium*, other non-*Mycobacterium* bacteria, and unassigned bacteria captured and sequenced from the sputum samples.

Mapping was then carried out to obtain as many reads of *Mycobacterium* as possible and eliminate the non-*Mycobacterium* reads. Reads were kept or eliminated based on how similar the query sequence was to the target sequence, i.e., the identity. This value depends on the reference sequence used, the length of the query, and the genomic edit distance (ED) allowed by the alignment algorithms. Genomic edit distance indicates the number of nucleotide edits (substitutions, insertions, and deletions) that would be required to transform the input read to the reference genome. Sequence mapping stringency or flexibility is closely related to the inclusion or elimination of reads which could belong to different taxonomic categories, based on the number of changes allowed.

The genomic variability of interest’s microorganism is determinant to select a specific ED value, highlighting the importance of avoiding default software settings since the optimal ED may differ depending on the dataset or organism of interest.

The SURPI software uses an ED value of 12 as a default parameter. Using an ED = 12 for mapping long sequences might be appropriate, but the alignment could be very lax in short ones, allowing sequences with many variations that could even belong to another contaminant bacteria. In contrast, an ED = 0 will be a perfect match, i.e., an identical sequence to the reference. However, this stringency value does not allow any relevant genetic variation to define drug resistance, virulence, or other *Mycobacterium* condition. Therefore, it was necessary to identify an intermediate value, lax enough to retain *Mycobacterium* reads with gene variants but stringent enough to eliminate sequences from other bacteria. Then, we evaluate two fundamental parameters to choose the best mapping condition: 1) we used two reference genome options, the whole genome of *Mycobacterium tuberculosis H37Rv* (NC_000962.3) and the custom gene panel, and 2) we tested different ED values for both reference genome options (Fig 1).

The reads obtained under these mapping conditions were re-aligned with the bacterial reference SURPI database to quantify the number of sequences classified as *Mycobacterium*, other non-*Mycobacterium* bacteria, and unassigned bacteria. The best mapping condition should choose a reference genome and an ED value that maximizes specificity and sensitivity, i.e., with the highest percentage of reads of *Mycobacterium* and the lowest for all other taxa (Fig 1).

#### 3rd step: Filtering with GATK for variant calling

Once the reads of other microorganisms were filtered, our objective was to perform a variant calling that minimizes bias, obtaining reliable results. First, the flow cell lines of each sample were sorted and merged, and duplicate reads were marked, then we used the Qualimap software to assess the presence of bias in sequencing and mapping artifacts, followed by a Mapping Quality filter (MAPQ <20) to remove potential erroneous alignments in the bam files of all samples (Fig 1).

Finally, we performed variant calling with the GATK software, including INDELs realignment, BQSR, individual and population variant calling. Lastly, we applied a strict hard-filtering [18, 21] and a post-filter to minimize false-positive variants (Fig 1).

The steps of INDELs’ realignment and BQSR require a database of known variants to reduce errors and refine the alignment of reads before the variant calling. Therefore, we constructed an *ad hoc* database, adapting the existing databases TB-Dream [24], PhyResSE [25], and ReSeqTB [26]. Our database included 1376 expected variants in 44 genes, annotated according to the positive strand in the whole *H37Rv* NC_000962.3 genome (S1 File). This database can be edited to include rare or particular variants of interest, which the software can easily identify.

Subsequently, the Haplotype Caller algorithm was used to perform individual variant calling and estimate the probability of SNPs and INDELs for each nucleotide in the genome. Although *Mycobacterium* is a haploid organism, sputum samples may contain mixed populations or strains transitioning from heterorresistance to complete resistance [27]. Therefore, we tested haploid and diploid modes for variant calling and found that only the diploid mode detected all the variants, including the heteroresistants previously identified by GeneXpert in some of our samples. Thus, the diploid mode was used from here on.

We then performed a population variant calling, considering all samples simultaneously to generate the genotype probabilities and detect the variants with low depth in a VCF file. The GATK hard-filtering was subsequently applied to remove or maintain population variants based on the following parameters for SNPs: QualByDepth (QD) <2.0; FisherStrand (FS) >60.0; RMS-MappingQuality <30.0; MappingQualityRankSumTest <-12.5; and ReadPosRankSumTest <-8.0; and for INDELs: QD <2.0; FS >200.0; ReadPosRankSum <-20.0) [18, 21].

To ensure quality control and reduce false-negative variants, we considered only samples with a high proportion of variant called, eliminating samples with a high proportion of non-called or missing variants. Summary statistics were thus performed on the hard-filtering results of the variant calling population to identify the cut-off of missing variants allowed to preserve the samples.

Finally, we applied an additional post-filter to the variants that passed the hard-filtering to obtain a more accurate and reliable result, considering only variants with SNPs or INDELs, i.e., those with alternate homozygous genotype 1/1 for complete resistance and heterozygous 0/1 or 1/0 for heterorresistance, in the case of subpopulations Then, we included only reads with a variant depth (DP) ≥10 and genotype quality (GQ) ≥30.

The *rrs* gene has conserved regions showing similarity between microorganisms, and therefore we decided to quantify the percentage of this gene’s variants to measure the potential contamination by other bacteria (Fig 1).

### Statistical analyses

Descriptive statistics and linear models were generated in the R programming language version 3.3.2 [28] to evaluate the alignment options and contrast them for each linear model by Fisher’s least significant difference tests. The Bonferroni *P*-value correction was applied, and a significance level of 0.05 over the adjusted *P*-values was used. In this context, alignment options sharing at least one letter (A, B, or C) were not considered statistically different. Furthermore, Venn diagrams were obtained to identify similarities or differences between the variants’ loci under each alignment option.

## Results

The bioinformatic pipeline proposed to analyze NGS data generated from TB clinical samples, selected first *Mycobacterium* sequences only, and then reliable variants. Sequences were filtered according to their quality. Those classified as human, viruses, or bacteria other than the *Mycobacterium* genus were eliminated. We then selected the variants by the following criteria: robustness according to the expected population variants, mapping quality, variant depth, and genotype quality. The results obtained by each step of our pipeline will be described as follows (Fig 1):

### 1st step: Input sequences with high-quality base calls

We obtained a total of 211,780,138 reads from 38 samples (theoretical depth of coverage 3426X). After the quality checkpoint, we eliminated 31.1% of reads and kept 145.8 million sequences with quality QV >30 and theoretical depth of coverage 2359X. Most reads were >130 bp in length, ranging from 70 bp to 134 bp (S1 and S2 Figs).

### 2nd step: Microorganisms identified and filtered by alignment with SURPI

Sequences aligning to the human genome (5.1% of all reads) and the virus database (0.04%) were eliminated. The resulting 138.4 million reads were aligned to the bacteria database for taxonomic identification (Fig 1 and S2 Fig).

#### Identification of the sequenced microbiota

**Fig 2:**
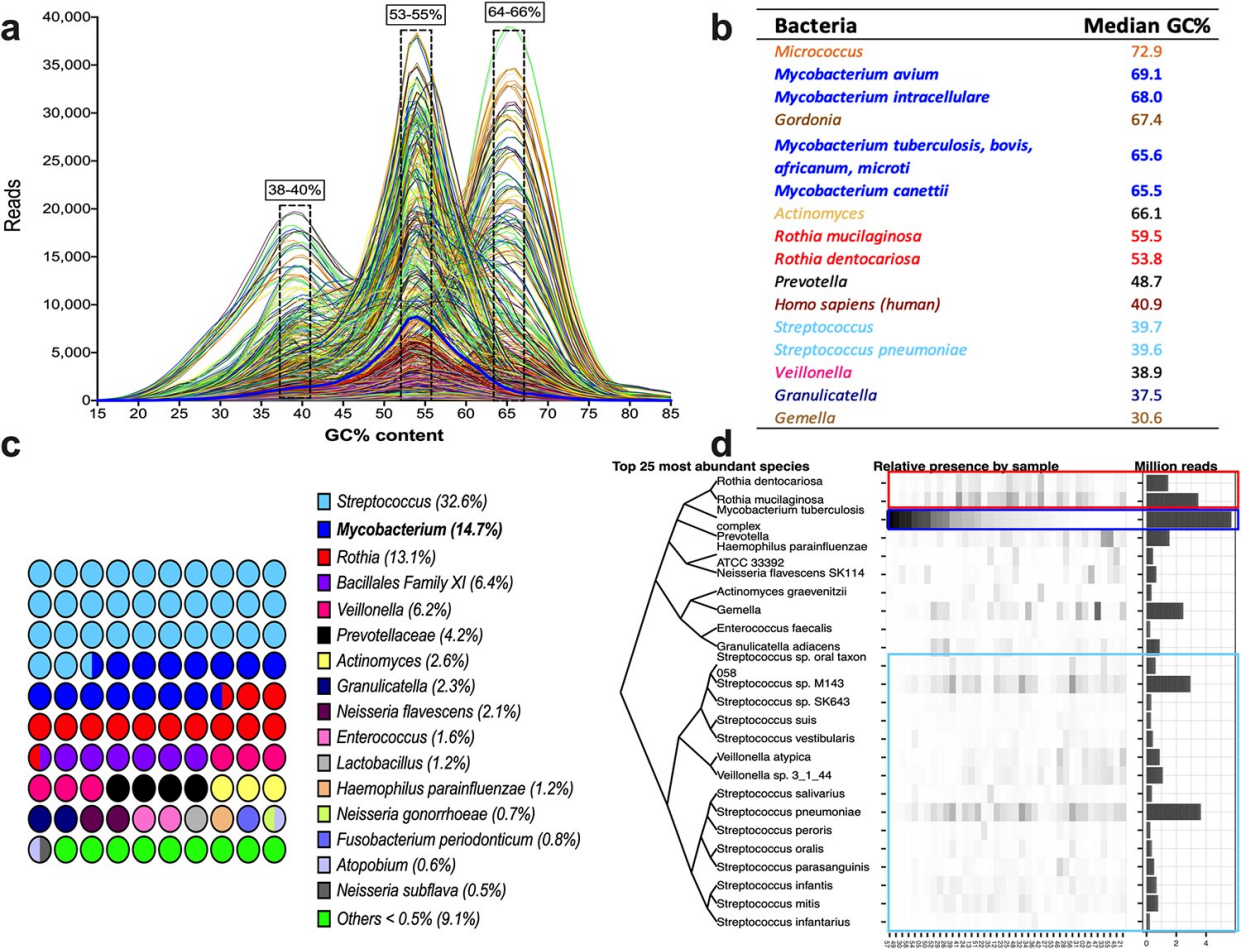
Reads by GC content and microbiota identified by SURPI after the alignment to the bacteria database. a) Distribution of reads of the 38 samples according to lines of the flow cell and percentage of GC-content. b) Median GC-content of the leading oral cavity and respiratory tract bacteria identified in the analyzed samples. c) Percentage of the main bacterial genera identified in all samples. d) Dendrogram showing the 25 most abundant species, as well as their relative and absolute presence by samples. The light blue rectangle corresponds to the *Streptococcus* genus, dark blue to *the Mycobacterium* genus, and red to the *Rothia* genus.

The analyzed sequences showed a trimodal distribution with peaks at 38–40%, 53–54%, and 65–66% of GC-content. The *Mycobacterium* genus has a GC-content of around 65.6%, and according to the SURPI assignation, only 14.7% of the total reads corresponded to the *Mycobacterium* genus, showing high heterogeneity between samples (Fig 2).

Among the unwanted bacteria, the most prevalent genera identified were *Streptococcus* (32.6%), mainly *S*. *pneumoniae* and *S*. *sp*. *M143* (Fig 2C and 2D, blue light rectangle), followed by *Rothia* (13.1%), predominantly *R*. *mucilaginosa* and *R*. *dentocariosa* (Fig 2C and 2D, red rectangle). *Rothia* genus sequences were abundant in samples with a low number of *Mycobacterium* reads (Fig 2D, blue, dark, and red rectangles). Other unwanted genera were *Veillonella* (6.2%), *Prevotellaceae* (4.2%), and *Actinomyces* (2.6%) (Fig 2C). Similarly, other bacterial species identified with a high number of reads were *Gemella* (*Bacillales Family XI*) (6.4%) and *Granulicatella adiacens* (2.3%) (Fig 2C and 2D). These bacterial genera represented 82.1% of all reads, and their GC-content matches the peaks shown in Fig 2A. *Streptococcus*, *Veillonella*, and *Granulicatella* have a GC-content around 38–40%, whereas *R*. *dentocariosa* has 53.8%, matching the first and second peaks. The third peak agrees with the *Mycobacterium* GC-content of 65.6%; however, *R*. *mucilaginosa* and *Actinomyces* have 59.5% and 67.4%, flanking the *Mycobacterium* peak (Fig 2A and 2B).

#### Filtering unwanted bacteria

We evaluated the best mapping option by aligning the 138.4 million sequences with the whole *M*. *tuberculosis H37Rv* genome or the custom gene panel, using ED values ranging from 12 to 0. The resulting sequences from each condition were re-aligned with the bacteria database for taxonomic classification (Fig 1).

**Fig 3:**
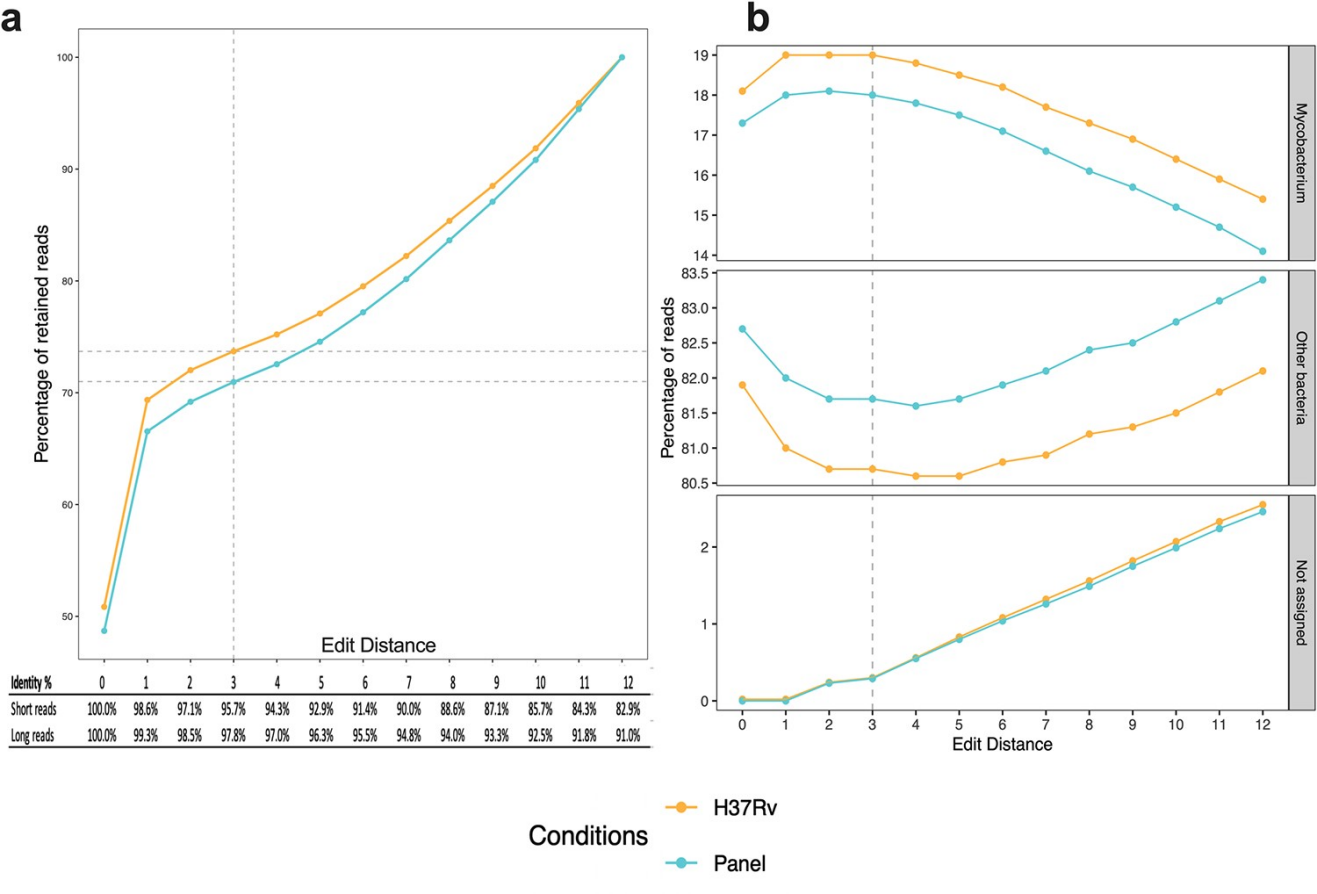
Alignment of reads with the reference genomes under each of the indicated edit distance values. The percentage of retained reads was calculated considering the total accumulated reads of *Mycobacterium*, other bacteria, and unassigned bacteria at each editing distance value. The alignment conditions were the whole *M*. *tuberculosis H37Rv* genome and the customized gene panel. a) Distribution of conserved reads after each mapping condition with the reference genomes and ED values from 0 to 12. Reads with more changes than the indicated ED values were removed. The identity value from each ED was calculated considering the shortest (70 bp) and the longest (134 bp) reads. b) Distribution of reads from each ED value: Top, percentage of *Mycobacterium* reads; Middle, percentage of other bacteria reads; and Bottom, percentage of unassigned reads.

The alignment with an ED = 12 retained the total number of reads, but the identity varies from 82.9% (58/70) in the shorter reads to 91% (122/134) in the longer reads (S1B Fig, Fig 3A). These values increase when the ED decreases, reaching 100% when the ED value is 0, but the total number of conserved reads decreased (Fig 3A). When mapping was performed with ED values >3, reads assigned to the *Mycobacterium* genus decreased, while reads from other types of bacteria or unassigned bacteria increased (Fig 3A and 3B). Selecting an ED value ≤ 3 eliminated around 30% of reads, but around 70% of the retained sequences showed 95.7 to 100% identity (Fig 3A and 3B).

Based on these results, we used a stringent ED = 3 to run our pipeline and performed comparisons with the lax default ED = 12. Using an ED = 3 still included reads from other contaminant microorganisms; however, these reads can be filtered in subsequent pipeline steps.

**Fig 4:**
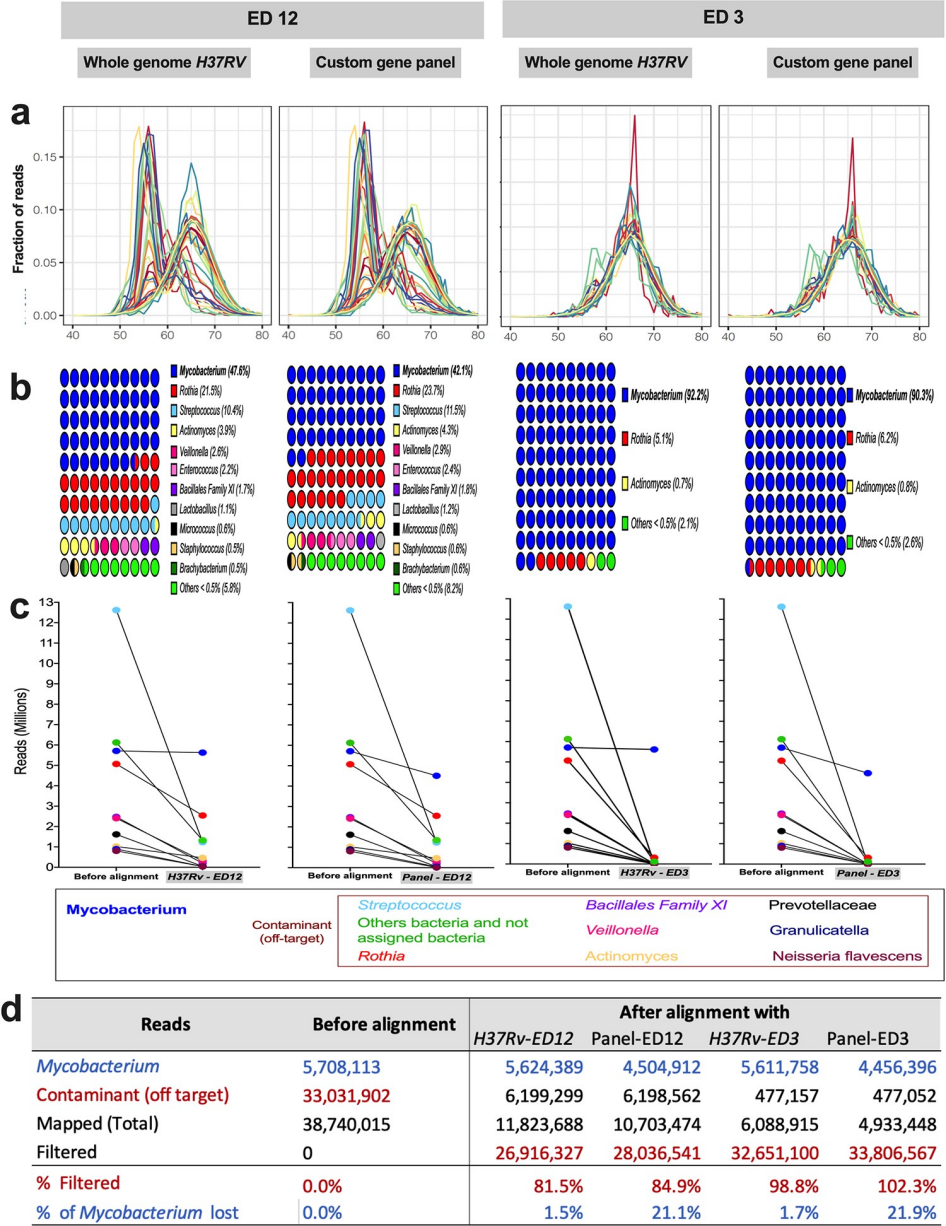
Microbiota identified under different mapping conditions and distribution of reads according to GC-content. Graphs showing the results after alignment with an ED = 12 (left) and ED = 3 (right), with the whole reference genome *H37Rv* or the *ad hoc* gene panel. a) GC-content distribution of the mapped reads, where each line represents a sample. b) Graph showing the percentage of the main bacterial genera identified in all samples. c) Graphs show the absolute number of reads assigned to the eight main bacteria genera before and after mapping under each alignment condition. d) Comparative table with *Mycobacterium* and contaminant reads, showing the percentages of filtered and deleted *Mycobacterium* sequences to evaluate the filtering efficiency during mapping. Alignment with an ED = 3 shows more than 100% filtered sequences because the stringent filter eliminated reads initially identified as *Mycobacterium*.

After mapping with the different alignment conditions, sequences’ GC-content showed a bimodal distribution when we used an ED = 12 and almost a unimodal distribution with an ED = 3 (Fig 4A). Reads belonging to *Streptococcus* decreased from 32% before mapping with the reference genomes to 10.4–11.5% after mapping using an ED = 12. These values descended to 0.1% with an ED = 3. Similarly, the number of *Veillonella* reads decreased from 6.2% to 2.6–2.9% when using an ED = 12 to 0.04% with an ED = 3 (Figs 2C, 4B and 4C). *Granulicatella* was initially identified with 2.3% reads; after mapping with an ED = 12 decreased to 0.4% and 0.001% with an ED = 3. The situation was similar with other bacteria with few counts (Figs 2C, 2D, 4B, 4C and S3 Fig).

The lower number of peaks by GC-content on the graph after alignments indicates reduced contamination (Fig 4A). For example, the peak at 38–40% GC-content was present before mapping but not after (Figs 2A and 4A). Most reads belonging to the peak at 53–55% GC-content were also removed by mapping with an ED = 3 (Fig 4A). Nevertheless, the contaminant reads were not completely removed. According to the taxonomic classification by SURPI, the main contaminant bacterial species identified were *Rothia mucilaginosa* and *Rothia dentocariosa* (S3 Fig), whose GC-content is around 59 and 54%, respectively (Fig 2B). This bacterial genus decreased from 23.7% by aligning with an ED = 12 to 5.1% with an ED = 3, representing around 0.3 million contaminant reads (Fig 4B and 4C).

Other bacterial genera filtered using an ED = 3 were *Actinomyces* and *Gemella*, which decreased from 4.4% to 0.8%, and from 1.8% to 0.03%, respectively (Fig 4B and S3 Fig). On the other hand, bacteria such as *Prevotellaceae* or *Granulicatella* genera initially had 1.6 and 0.9 million reads but presented <65 reads after filtering. These numbers clearly showed that the stringency condition using an ED = 3 produced a better filtering performance of unwanted bacteria than with an ED = 12.

The peak of GC-content around 65–66% was preserved and is coincident with the median GC-content of *Mycobacterium*. Before mapping, *Mycobacterium* reads were 14.7% (Fig 2), increasing to 42–47% after alignment to the reference genomes with an ED = 12 and 90–92% with an ED = 3. It is important to mention that the absolute number of reads assigned to the *Mycobacterium* genus was not substantially affected by the filtering process (Fig 4C); accounting for 5.7 million reads before mapping and 5.6 million after alignment with the *H37Rv* reference genome, i.e., <2% was lost (Fig 4D).

### 3rd step: Variants called with GATK

Variant calling of each alignment option was analyzed by comparing variant characteristics such as depth (DP), quality (QUAL), mapping quality (MQ), and the number of variants filtered. Linear models were generated considering the reference genomes (whole *H37Rv* genome or *ad hoc* gene panel) and two ED values (ED = 12 as the default condition, or ED = 3 as our stringent filtering proposal).

**Fig 5:**
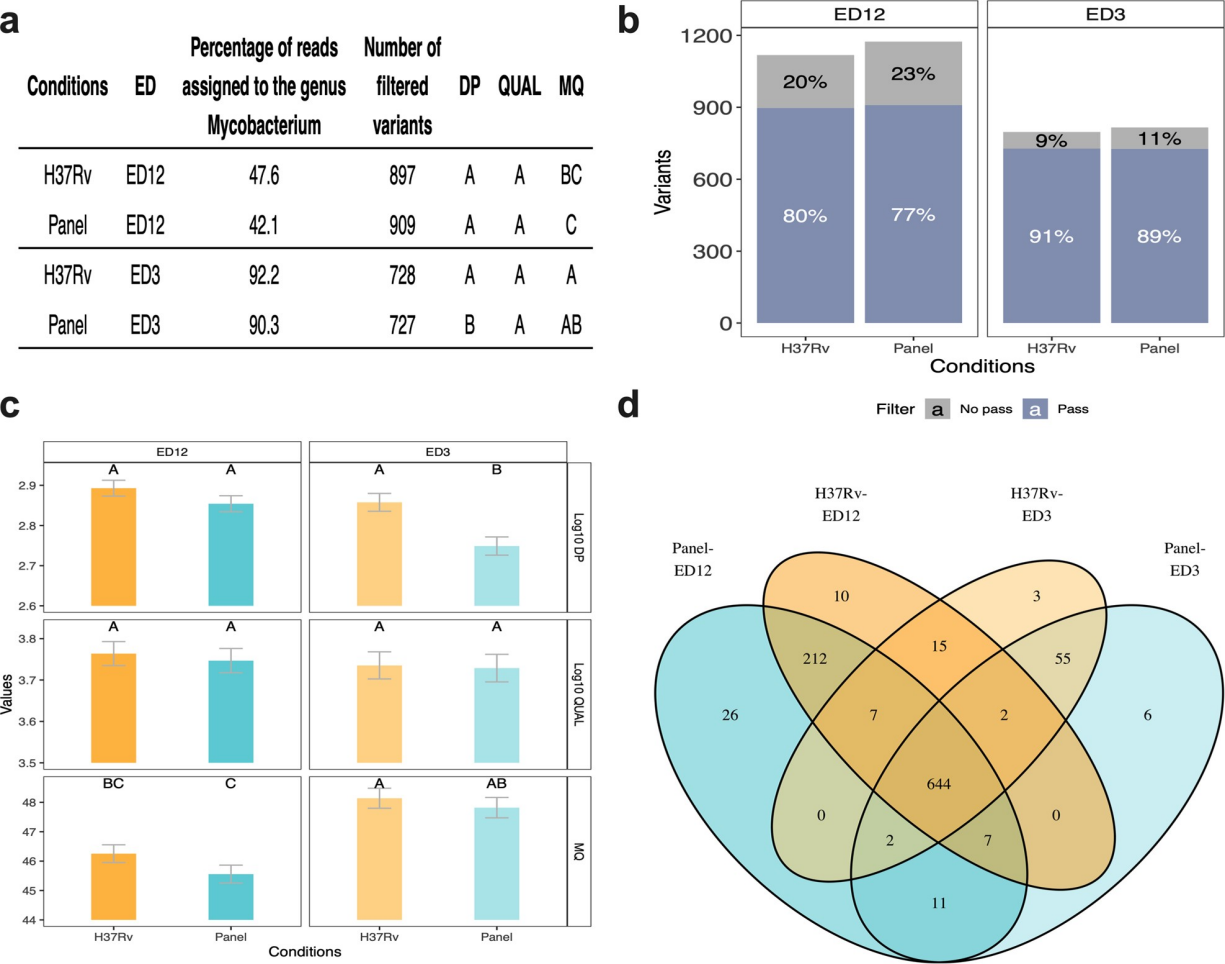
Comparison of *Mycobacterium* variants obtained under the indicated alignment conditions. a) Comparative table of results obtained under different alignment conditions. b) Percentage of variants that passed or did not pass the hard-filtering at each alignment condition. The Y-axis scale indicates the absolute number of reads. c) Characteristics of the variants such as depth (DP), quality (QUAL), and mapping quality (MQ) under each alignment condition. A, B, C, AB, and BC correspond to Fisher’s test results with the least significant difference. d) Venn diagram comparing the variants according to their loci between alignment conditions.

The sequences aligned with ED = 12 showed more variants than with ED = 3, but many of these did not pass the hard-filtering (Fig 5A and 5B). The proportion of reads assigned to the *Mycobacterium* genus was less than half, increasing the possibility of generating false-positive reports (Fig 5A). According to the linear models, variants that passed the hard-filtering showed a similar PD between alignment conditions, slightly lower when mapping with the gene panel using an ED = 3 (Fig 5A–5C). Regarding quality, variants identified by the four conditions tested showed similar QUAL values. However, the MQ values differed between groups. The best quality was obtained aligning with the *H37Rv* genome at ED = 3, while the lowest quality value was observed after aligning with the gene panel at ED = 12 (Fig 5A–5C).

The Venn diagram shows greater differences between variants’ numbers when comparing the ED parameters (3 or 12) than reference genomes (whole genome or gene panel). For example, alignments carried out with an ED = 12 showed 229 and 245 variants not identified with an ED = 3 when the alignments were performed using the whole reference genome or the gene panel, respectively. In contrast, alignments with the whole genome showed only 25 variants not identified after the alignment with the gene panel at ED = 12 and ED = 3. The alignments with either reference genome shared 96.6 and 97.2% variants. Most of these 25 different loci belonged to the PE/PPE family and ribosomal genes that should be eliminated or analyzed independently (Fig 5D).

**Fig 6:**
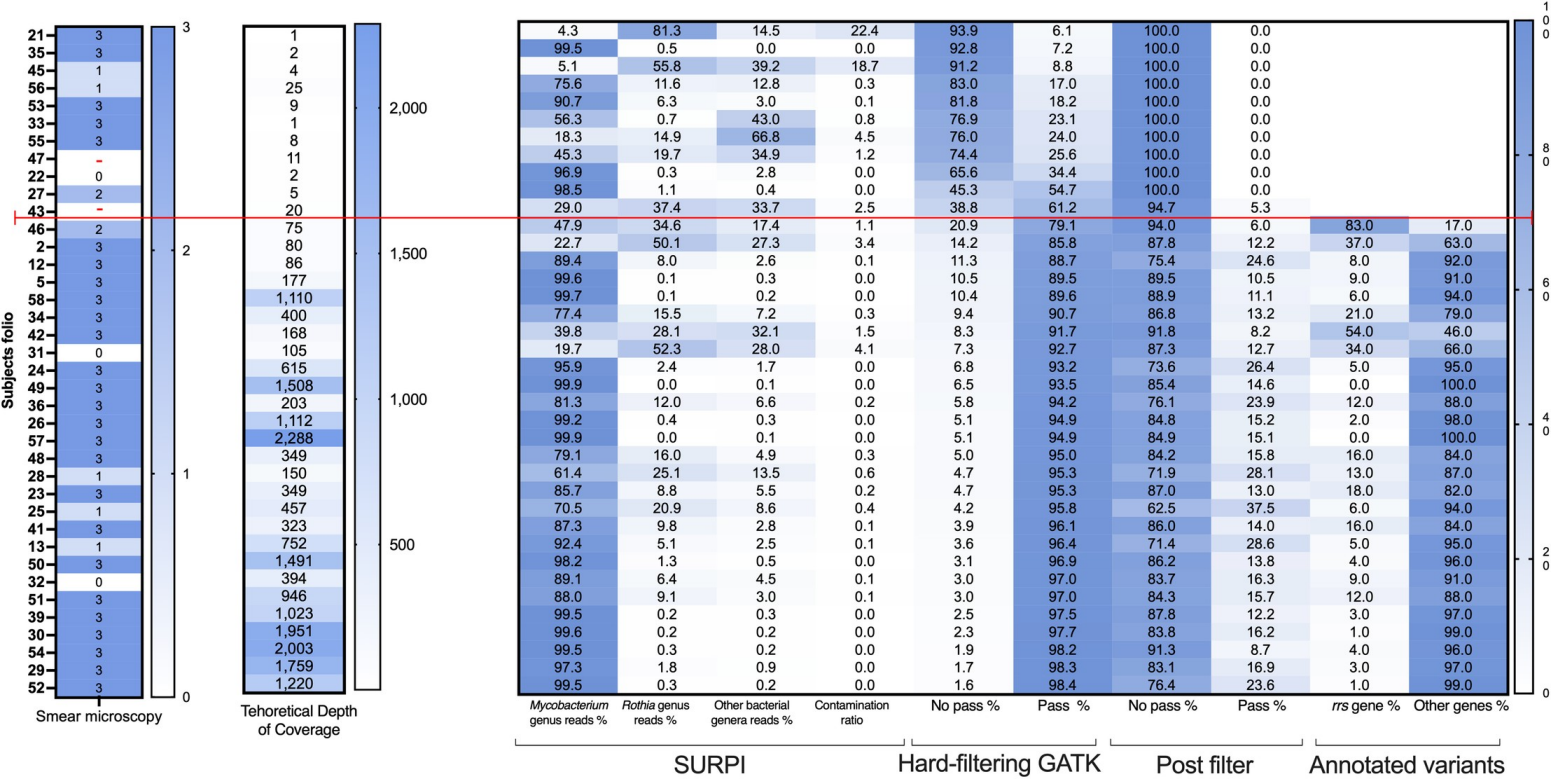
Heat maps of the samples according to the indicated filter steps. The samples are ordered according to the percentage of missing variants. The results of acid-fast bacilli smear microscopy, theoretical depth of coverage, identification by SURPI, and percentage of variants that passed or did not pass the GATK hard-filtering and post-filter, as well as the *rrs* gene variants percentage, are shown. The red line divides the samples that passed (below) or did not pass (above) the hard-filtering. The percentage of variants that passed the post-filter is lower than those that passed the hard-filtering because only variants with SNPs or INDELs were selected in the post-filter.

The proposed pipeline managed to filter reads and variants of several unwanted microorganisms from the 38 sputum samples. However, the bacillary load, number of *Mycobacterium* reads, and contamination levels of each sample were different (Fig 6). Some samples had low reads’ depth and low contamination; however, most of these sequences belonged to *Mycobacterium*. In contrast, another group of samples presented a good depth of *Mycobacterium* reads but also high contamination. In both cases, the analysis through the pipeline eliminated off-target reads, keeping a good number of *Mycobacterium* reads that supported the variant calling. A third case was observed in samples with low depth of *Mycobacterium* reads and high contamination by *Rothia* and other bacteria; therefore, few *Mycobacterium* reads supported the variant calling. (Fig 6 and S3 Fig).

The samples with low depth coverage of *Mycobacterium* reads had higher number of variants that did not pass the hard-filtering, i.e., missing variants. The distribution of these variants by sample and genotype is shown in the two left panels of S4 Fig. Most samples had 79.1 to 98.4% variants that passed the hard-filtering (Fig 6, Pass % column below the red line, hard-filtering condition); i.e., a maximum of 20.9% of missing variants. However, in another group with eleven samples, including the two negative controls (samples 47 and 43), we observed 38.8 to 93.9% of missing variants (Fig 6, No Pass % column above the red line, hard-filtering condition). These samples showed a theoretical depth of coverage ≤ 25X, less than 41,000 *Mycobacterium* reads, and only between 6.1 and 61.2% of the variants called. (Fig 6, Pass % column above the red line, hard-filtering condition). Therefore, we eliminated these eleven samples with more than 25% of missing variants and performed a new population variant calling (S4 Fig, filtered panels).

On the other hand, samples with a contamination ratio >1, such as samples 2, 31, 42, or 46, could still generate false positive variants, even when they passed the hard-filtering. Therefore, our pipeline introduces a post-filter to deal with this issue. For instance, sample 2 had a theoretical depth of coverage of 80X, but only 22.7% of reads were *M*. *tuberculosis*, and 50.1% belonged to the *Rothia* genus. After the variant calling, 85.8% passed the hard-filtering and, of this total, only 12.2% passed the post-filter. We also observed that 37% of these variants corresponded to the ribosomal *rrs* gene (Fig 6), suggesting that these could represent potential contamination and should be treated cautiously. The opposite scenario can be exemplified by sample 52, which had a theoretical coverage of 1220X, with 99.5% of reads assigned to *Mycobacterium* and almost null contamination. After the variant calling, 98.4% of reads passed the hard-filtering, of which 23.6% passed the post-filter, with only three variants belong to the *rrs* gene.

In summary, our results show that the samples with low contamination lose very few sequences during the filtering steps of the bioinformatic process, while those with almost null contamination, even those with low bacillary load, were filtered mainly in the final steps. In contrast, more contaminated samples lost sequences in several steps until they finally presented few reads of *M*. *tuberculosis*. Therefore, it is important to begin the analysis with many sequences from each sample to mitigate the losses by contamination. The steps included in the proposed pipeline eliminate sequences and variants unrelated to *Mycobacterium*, even in samples with different degrees of contamination or bacillary load.

## Discussion

The use of NGS for basic and translational TB research has been a fast-growing field. However, regarding the bioinformatics analysis, it is generally assumed that exclusively *Mycobacterium* reads and variants will be obtained when mapping with the reference genome under the pre-established parameters of the aligners. Our study demonstrates that DNA sequences from other contaminant bacteria present in sputum samples were captured and sequenced, even when we used a specific DNA extraction method, a target enrichment method designed for *Mycobacterium*, and a bioinformatics pipeline that aligned the sequences to the reference genome under default conditions.

We observed considerable bacterial diversity among sequences, as indicated by reads with different GC-content [29]. The genera *Streptococcus*, *Rothia*, *Actinomyces*, and *Veillonella*, were identified by SURPI, mainly when a lax genomic edit distance (ED = 12) was used in the alignment. These bacteria, which naturally inhabit the respiratory tract and oral cavity, have been found in other studies analyzing TB samples [27, 30]. A recent study identified contaminant reads using Kraken as a taxonomic classifier of TB-WGS sequences from different published studies. *Streptococcus*, *Rothia*, and *Actinomyces* were detected, but *Veillonella* was only present in studies based on the capture sequencing method [31], similar to the present study. The reads from these unwanted bacteria can generate false-positive results. We identified >200 different variants when mapping with an ED = 12 which could belong to other bacterial genera.

This study’s key to eliminating most unwanted microorganisms was to include a stringent filtering condition in SURPI using an ED = 3, allowing an identity of 97.8%. This condition implied that only around 2% of the genetic variation was allowed during the *Mycobacterium* reference genome alignment. These changes include variations due to specific bacterial conditions such as resistance, virulence, changes related to subspecies, among others [32].

The identity recommended from our results (97.8%) is similar to that used by Galo and col (97%) to identify DNA contamination in bacterial sequencing experiments [31]. Both values are more stringent than the 96% set by default in alignments with the BWA tool [33]. It is important to note that this high specificity does not exclude from the analysis new rare variants belonging to the microorganism of interest, as long as they have a good mapping quality and good variant depth. These two requirements are essential to consider them as highly reliable to pass the hard-filtering and post-filter.

The edit distance can usually be modified as a number or a percentage in different mapping software. However, we should consider the length of the read, the genomic variability of the microorganism of interest, the correct commands, and the options for this parameter in the aligner used. Avoiding sequences with a high percentage of changes during mapping will remove sequences from other microorganisms and improve the specificity of variants of interest.

Regarding the reference genome options for mapping, we consider the whole *H37Rv* genome a better and more efficient alternative than the gene panel, mainly because the *H37Rv* genome showed slightly higher mapping quality and depth. The use of the whole genome probably means that all reads (including those off-target) align more reliably and freely to the corresponding region, without constraints or bias of mapping exclusively to the regions bioinformatically designed. Mapping the reads with the whole *H37Rv* reference genome with an ED = 3 increased the percentage of *Mycobacterium* reads from 14.7 to 90–92%. This condition produced mapping qualities >48, eliminating almost 99% of contaminant reads and losing only <2% of *Mycobacterium* sequences.

Our bioinformatic analysis proposal consists of several steps. The initial alignment and filtering steps with SURPI eliminated many sequences from contaminant microorganisms, only keeping reads highly similar to *Mycobacterium*. For example, some distantly related bacteria such as *Streptococcus* were easily eliminated in the initial steps by stringent mapping with an ED = 3. On the other hand, *Rothia* seems so genetically close to *Mycobacterium* that some reads passed these filters. However, the subsequent use of the GATK hard-filtering increased the variant calling specificity for each sample, yielding truly positive variants. According to the population calling, sequences from other bacterial genera displayed different variants than those expected for *Mycobacterium*. This condition was verified by the negative controls included in the study.

According to previous reports, we identify *Rohia* in TB sputum samples, which has been considered a co-pathogen [27, 30, 34]. This finding highlights how easy it is to report false-negative variants when *Rothia* is not adequately eliminated and raises questions about its function in the pulmonary microbiome of patients with this disease. In fact, *Rothia* represented the greatest elimination challenge. However, we found a broad variability of contamination in the samples, regardless of bacillary load. In this regard, removing contaminant sequences and other microorganisms’ variants was possible even in samples with negative smears but positive culture, as long as there was at least a depth of coverage of 25X and 41,000 *Mycobacterium* reads. This condition exhibits the importance of deep sequencing in clinical samples, filtering reads at each pipeline step and maintaining a good final depth per variant (DP ≥10).

We should consider that the methodology used in this NGS study was target enrichment through capturing genomic regions of interest by hybridization to biotinylated probes. Commercial targeted-NGS protocols consider different DNA fragmentation methods and alternatives for DNA fragment lengths, probes, density, and layouts around target regions. These conditions can contribute to inconsistent results and the presence of off-target reads to a greater or lesser degree [35]. Although target enrichment methodology optimizes costs, we should consider the proportion of off-target reads generated [31, 36].

Other reasons for off-target reads could be related to the specific regions included in a targeted panel since the designed probes could hybridize with orthologous genes or conserved loci of other bacteria present in clinical samples. This aspect shows the relevance of properly selecting the genes or gene families included in the analysis to minimize contamination through genetic similarity. Additionally, we should verify that only sequences of the interest microorganism will be obtained, even when target enrichment techniques are used. Our results highlight the importance of using an adequate pipeline to filter non-targeted microorganisms to avoid false-positive results, by adjusting the genetic editing distance during the alignment, according to the length of the sequences obtained and the expected genetic microorganism of interest variability.

The use of clinical samples in TB research is always important; however, it has inherent limitations. We are aware that we only used negative controls in this study, and it would be desirable to include pure positive controls of *Mycobacterium*, such as a guaranteed sample from a commercial vendor or pure *M*. *tuberculosis* cultures from positive clinical samples. Nevertheless, contamination by off-target reads has also been identified in sequences from pure cultures of TB isolates [31]. Moreover, sequencing from biological samples such as sputum allows identification of *M*. *tuberculosis* subpopulations, commonly lost during culture.

## Conclusions

Our bioinformatic pipeline aimed to generate a strategy to analyze targeted-NGS data from clinical samples of *Mycobacterium tuberculosis* and eliminate off-target reads to reduce the number of false-positive variants. Our strategy was to perform the analysis, beginning with many reads from deep sequencing and targeted enrichment methods to achieve high specificity at two levels: sequences and variants. Specificity at the level of *Mycobacterium* sequences was achieved by mapping with the whole *H37Rv* genome using a genomic edit distance of 97.8% (ED = 3), which removed 98.8% of the off-target sequences. We accomplished specificity at the variant level by using the variant calling and filters of GATK, with an additional post-filter that eliminated more than 200 potentially false variants. This pipeline produced reliable results with high-quality reported variants. Furthermore, by identifying the off-target reads, we described the microbiome of clinical TB samples that could be captured by target enrichment, sequenced, and identified during the mapping under default parameters (ED = 12). These results showed the consistent presence of the genera *Streptococcus*, *Rothia*, *Actinomyces*, and *Veillonella*.

This bioinformatic strategy uses free software and could be applied to whole-genome sequences or gene panels, offering a valuable tool for heterorresistance identification of importance for diagnosis and treatment choices. We do not recommend using default parameters during mapping and encourage determining the best condition for each microorganism of interest. This adjustment will help with the exclusion of contaminant sequences, improving the report of true variants. We also emphasize the importance of designing a bioinformatic analysis that integrates computational and biological knowledge to obtain better results.

The code developed in this research could be found in a GitHub repository (**https://github.com/kachelo/NGS-analysis-from-TB-sputum**) for future references.

## Supporting information

S1 File Database of known variants of Mycobacterium tuberculosis. (VCF)

S1 Fig Data pre-processing. a) Raw reads and mean quality value across each base position. b) Distribution of fragment sizes (read lengths) after trimming. c) Mean quality value across each base position in the reads after trimming. d) Number of reads with average quality scores after trimming.
(TIF)

S2 Fig Number of reads after each pipeline step. a) Countdown of reads (continuous line) and theoretical depth of coverage in each step (dash line), from the original data to the alignment with the reference genome *H37Rv*, using an edit distance of three. b) Percentage of reads captured by Sure Select probes according to their classification by SURPI.
(TIF)

S3 Fig Panel of dendrograms shows the 25 most abundant species with their relative and absolute presence in each sample. We used genomic edit distances of 12 and 3 to align with the *H37Rv* genome and gene panel.
(TIF)

S4 Fig Quality distribution of samples and variants. Tile plots where rows represent population variants reported by GATK, samples are represented in columns, and color represent the genotypes: 0/0 (wild type in red), heterozygote variant 0/1 (green), or 1/0 (cyan), homozygote variant 1/1 (purple), and data not available or missing variants (NA or./. in gray). The variants (rows) are ordered according to the allele number from top to bottom. Samples (columns) are ordered by their NA content, leaving uninformative samples to the right. The whole picture is composed by the univariate density plot for the corresponding rows/columns of the Total (left) and Filtered (right) set of samples, obtained after the alignment to the whole *Mycobacterium tuberculosis* genome, with a genomic edit distance of 12 (top) or three (bottom).
(TIF)
