# Supplementary figures and images for: A bioinformatics pipeline for Mycobacterium tuberculosis sequencing that cleans contaminant reads from sputum samples
Source: PLoS One. 2021 Oct 26;16(10):e0258774. doi: 10.1371/journal.pone.0258774 (PMC8547644; doi:10.1371/journal.pone.0258774)

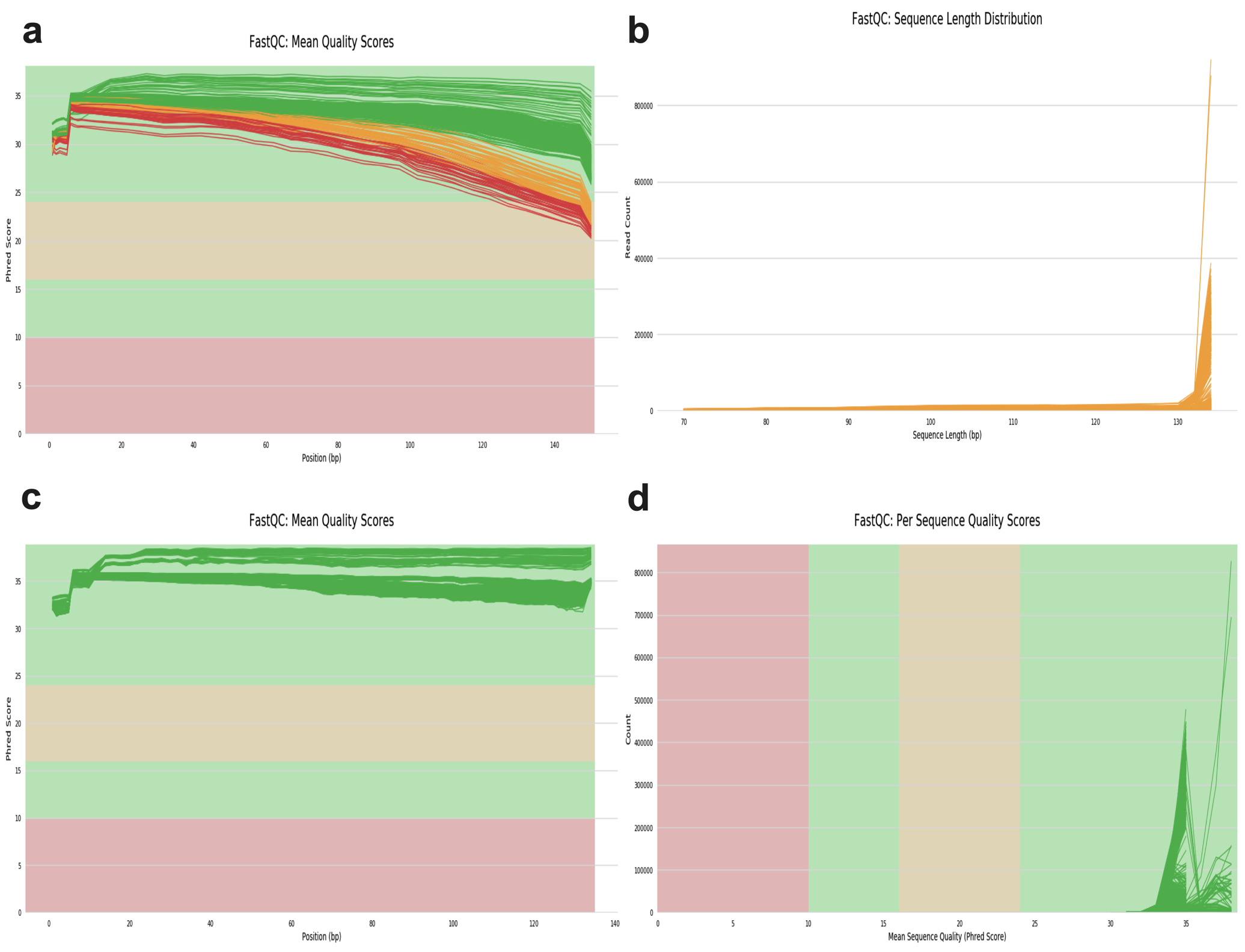

Supplement: S1 Fig — a) Raw reads and mean quality value across each base position. b) Distribution of fragment sizes (read lengths) after trimming. c) Mean quality value across each base position in the reads after trimming. d) Number of reads with average quality scores after trimming. (TIF) [file pone.0258774.s002.tif]

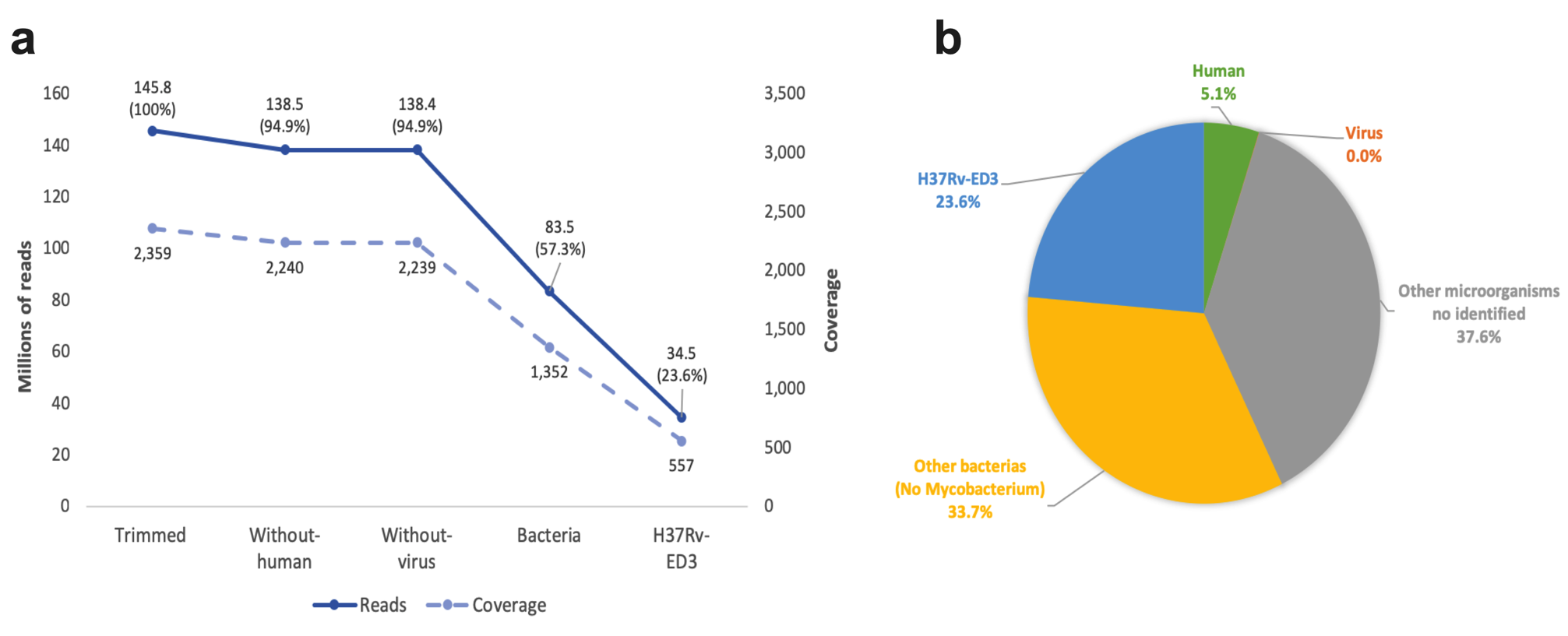

Supplement: S2 Fig — a) Countdown of reads (continuous line) and theoretical depth of coverage in each step (dash line), from the original data to the alignment with the reference genome H37Rv, using an edit distance of three. b) Percentage of reads captured by Sure Select probes according to their classification by SURPI. (TIF) [file pone.0258774.s003.tif]

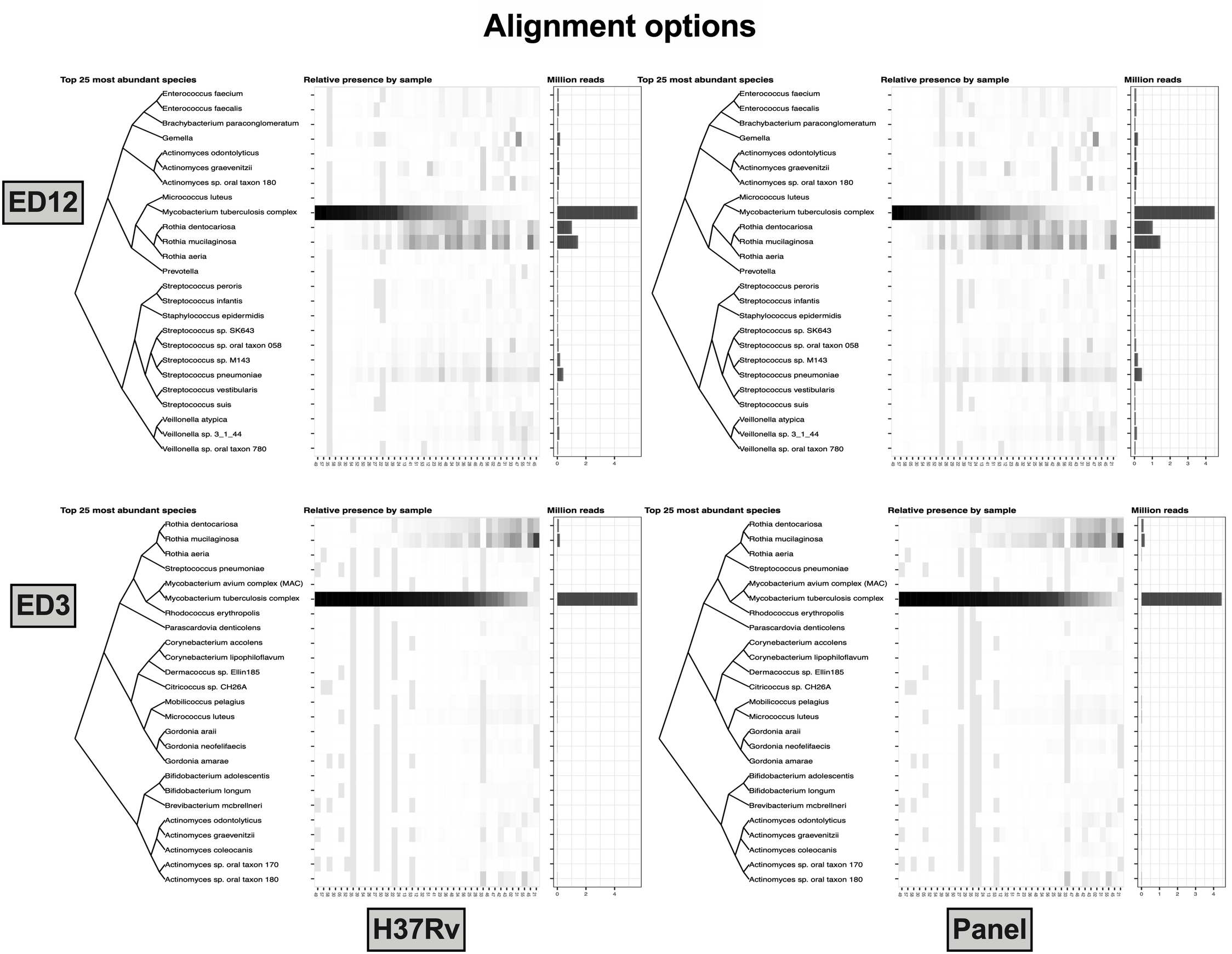

Supplement: S3 Fig — We used genomic edit distances of 12 and 3 to align with the H37Rv genome and gene panel. (TIF) [file pone.0258774.s004.tif]

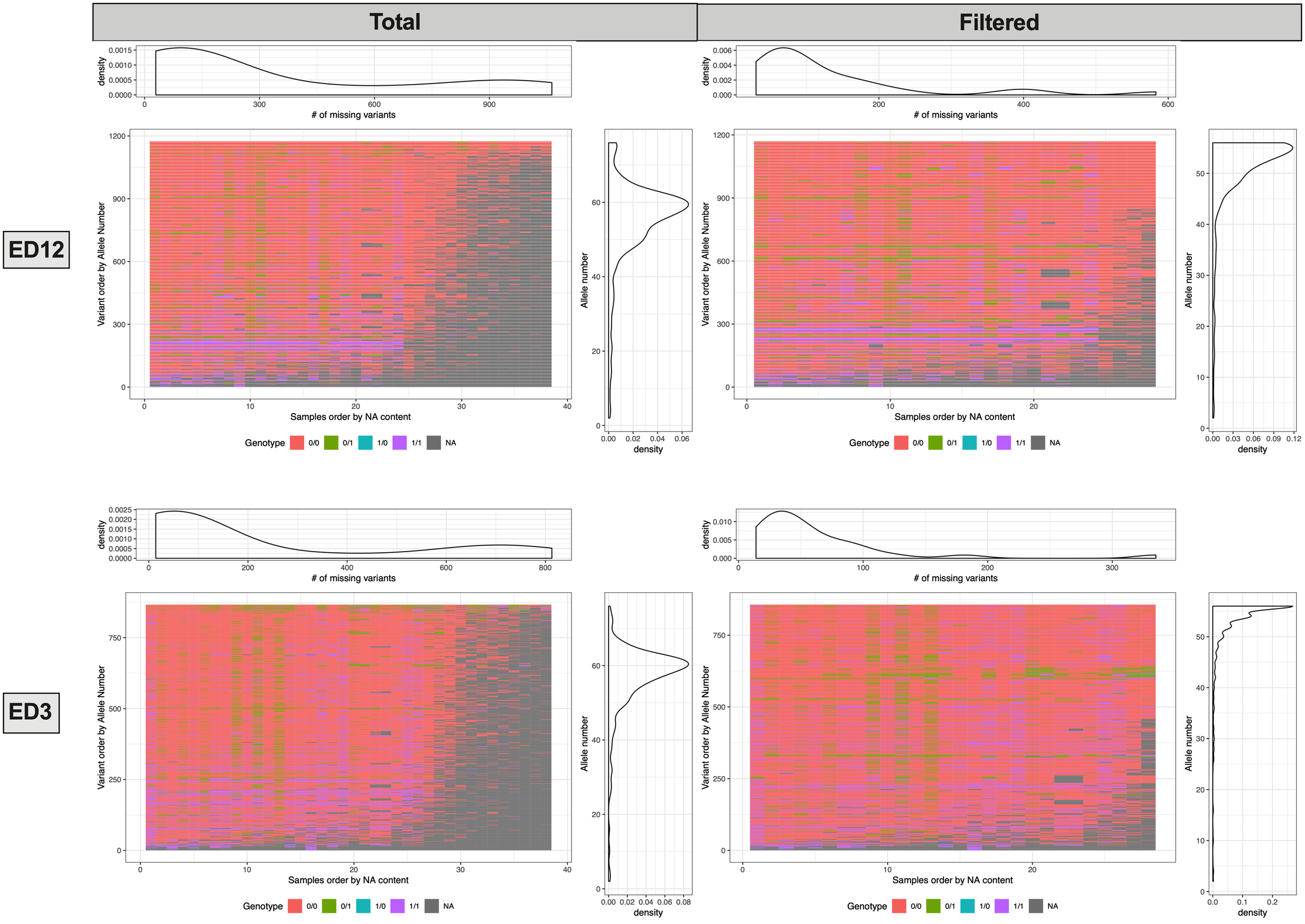

Supplement: S4 Fig — Tile plots where rows represent population variants reported by GATK, samples are represented in columns, and color represent the genotypes: 0/0 (wild type in red), heterozygote variant 0/1 (green), or 1/0 (cyan), homozygote variant 1/1 (purple), and data not available or missing variants (NA or./. in gray). The variants (rows) are ordered according to the allele number from top to bottom. Samples (columns) are ordered by their NA content, leaving uninformative samples to the right. The whole picture is composed by the univariate density plot for the corresponding rows/columns of the Total (left) and Filtered (right) set of samples, obtained after the alignment to the whole Mycobacterium tuberculosis genome, with a genomic edit distance of 12 (top) or three (bottom). (TIF) [file pone.0258774.s005.tif]
